# fiReproxies: A computational model providing insight into heat-affected archaeological lithic assemblages

**DOI:** 10.1371/journal.pone.0196777

**Published:** 2018-05-16

**Authors:** Andrew C. Sorensen, Fulco Scherjon

**Affiliations:** Human Origins Group, Faculty of Archaeology, Leiden University, Leiden, The Netherlands; Institut Català de Paleoecologia Humana i Evolució Social (IPHES), SPAIN

## Abstract

Evidence for fire use becomes increasingly sparse the further back in time one looks. This is especially true for Palaeolithic assemblages. Primary evidence of fire use in the form of hearth features tends to give way to clusters or sparse scatters of more durable heated stone fragments. In the absence of intact fireplaces, these thermally altered lithic remains have been used as a proxy for discerning relative degrees of fire use between archaeological layers and deposits. While previous experimental studies have demonstrated the physical effects of heat on stony artefacts, the mechanisms influencing the proportion of fire proxy evidence within archaeological layers remain understudied. This fundamental study is the first to apply a computer-based model (fiReproxies) in an attempt to simulate and quantify the complex interplay of factors that ultimately determine when and in what proportions lithic artefacts are heated by (anthropogenic) fires. As an illustrative example, we apply our model to two hypothetical archaeological layers that reflect glacial and interglacial conditions during the late Middle Palaeolithic within a generic simulated cave site to demonstrate how different environmental, behavioural and depositional factors like site surface area, sedimentation rate, occupation frequency, and fire size and intensity can, independently or together, significantly influence the visibility of archaeological fire signals.

## 1. Introduction

The number of anthropogenic fires lit in the past does not possess a one-to-one relationship with the present evidence attesting to these fires; that is, not every campfire leaves a durable and recognisable footprint in the archaeological record, which makes characterizing the frequency and nature of fire use by prehistoric humans notoriously difficult (e.g. [1]). This is largely due to the fact that so many different variables present within the *life-cycle* of a hearth can potentially factor in on the probability that the evidence of a fire will preserve [2, 3]. These include not only the myriad taphonomic factors working towards the post-depositional destruction of these traces (often exacerbated with greater antiquity) [4–8], but also local conditions at the time of burning (e.g. wet or dry substrate, wind speed) and human choices regarding fuel selection (i.e. wood, shrubby material, grass and/or bone) and hearth function (e.g. for cooking, warmth or lighting), size and duration. This inherent complexity has led to the implementation of a wide range of analytical methods attempting to identify anthropogenic burning in the archaeological record, including the identification and quantification of fire proxy evidence like heated lithic materials [9–13] and charred/combusted bone [14–16], soil and sediment micromorphology [2, 17–21], microcharcoal analysis [22–24], archaeomagnetism [25–27] and various forms of spectral analyses [28–31] and chemical characterisation [32–34] of fire affected materials and sediments. Added to this are numerous experimental studies testing the behaviour of both wood [35–37] and bone [38] fuels and the effects of heat on underlying substrates and artefacts therein [3, 39–45].

This study focuses on the common practice of using heated lithic remains (primarily siliceous raw materials introduced by humans via stone tool production) as a proxy for prehistoric fire use, where percentages of these artefacts are used to reconstruct the relative amount of fire use between archaeological layers or sites, as well as to infer probable locations of ancient hearths [9, 10, 46–48]. Heated lithics are usually identified on the basis of a series of macroscopically visible physical alterations. Changes in colour (artefacts often taking on a more reddish hue due to the presence of small amounts of iron) generally occur between 250–300°C [12, 49, 50], depending on the raw material and length of time exposed to these temperatures. Sergant and colleagues [12] noted these changes may not become readily apparent until a temperature of around 300°C is attained, and this was likely due to the heat exposure times or temperatures are sustained at the lower temperatures for a sufficiently long period of time [50]. Heating to temperatures above 350°C give the surface of some siliceous materials a more shiny lustre [51]. Overt thermal alterations in the form of cracking, crazing and thermal spalling (or ‘potlidding’) often begin to occur at temperatures above 400–500°C [50, 52], while obvious mass loss and whitening caused by calcination tends to indicate temperatures above 600–700°C [53, 54]. These heated lithic remains are either introduced into a fire while it is burning (e.g. as an errant flake during flint knapping or kicked in accidently while passing by), or present on the surface or within the sediment underlying a fire as relics from previous occupants of the site [42, 45, 55]. Given the generally low percentages of heated lithic artefacts observed on single occupation sites (e.g. [56–58], it appears that—save for some special circumstances where it appears large numbers of lithics have been purposely dumped into active hearths [12, 59]—relatively few artefacts are introduced directly into a burning fire. Therefore, it is our assertion that the heating of extant lithics in the substrate directly below hearths during subsequent occupations of a site is the primary driver of inflated percentages of fire proxies within archaeological deposits. Such fire proxy evidence is particularly important when hearth features or other less-durable direct evidences of burning (i.e. charcoal, ash and/or heated sediments) are lacking, and can also include faunal remains [16, 42] or stones occurring naturally in the substrate [60, 61].

It is generally assumed by researchers concerned with archaeological fire use that higher proportions of heated lithics indicate a higher degree of fire use on site, while very low percentages are assumed to indicate fire was either very infrequent or altogether absent [9, 10]. However, while these assumptions appear to be logical, it has recently been argued that various factors like prevailing climatic conditions, the position of an archaeological site in the landscape, and the nature of fire use on a site can drastically influence the number of fire proxies produced, even if fire use is a consistent feature between different periods of occupation [62]. Here, we utilize a computer-based model entitled 'fiReproxies' (R being the computer language used to code our simulation) to assess the potential effects of site size, sedimentation rates, fire size and intensity (i.e. fire duration and temperature), and site use intensity (i.e. the frequency, duration and nature of site use based on mobility patterns) on the generation of fire proxy evidence over time within a hypothetical Neandertal cave site. The results have major implications for how we interpret fire proxy data and stress the importance of understanding the roles the environment, site-formation processes and human choice play in the production and preservation of fire proxy evidence.

### 1.1. Evaluation of the assumptions regarding fire use parameters

The conclusions drawn by Sorensen [62] about the presence of relative quantities of fire proxy data between different archaeological contexts rely on a number of assumptions regarding the influences of human behaviour, geographical setting and depositional environment on fire use. We implemented the model to assess some of these assumptions by addressing the following hypotheses:

1. **Occupation surface size and shape**. Smaller living surfaces will increase hearth placement redundancy (the tendency of hearths to cluster) and, this will increase the frequency of heated lithic artefacts within a layer due to the elevated chances of fires being built over extant lithic scatters. Conversely, larger living surfaces will result in less hearth placement redundancy and, therefore, lower resultant percentages of heated lithics. The shape of the occupation surface may also influence the number of fire proxies produced.
2. **Relative placements of hearths and lithic scatters**. It is possible that hearth placement redundancy is higher when previous hearth locations are visible to subsequent groups, resulting in stacked or closely overlapping hearths. It is assumed that a visible hearth remnant would not only act as a focal point in the cave [63], but one with the perceived affordance of a good location to place a hearth since this location was selected previously [64–67]. Hearth placement redundancy becomes "less perfect" (i.e. farther away from the centroid of the previous hearth) as the time between occupations increases, allowing for more mobile ash deposits to be laterally dispersed, thus potentially obscuring the original footprint of the hearth. Assuming flintknapping activities are conducted near the fire (cf. [68]), hearth placement redundancy will increase the possibility for subsequent fires to be placed atop previous lithic scatters.
3. **Number of occupations per layer**. With every additional occupation of a site within an archaeological layer, the opportunity for incidentally heating lithics from a previous occupation increases. Therefore, layers with higher numbers of occupations will have higher percentages of heated lithics.
4. **Nature and number of fires**. Increasing the size, temperature and/or duration of a fire, as well as the number of hearths per occupation, will result in an increased capacity to heat underlying lithics, therefore increasing relative percentages of fire proxies.
5. **Number of lithics per occupation**. Increasing the number of lithic scatters deposited during an occupation would, in theory, increase the probability that a fire in a subsequent occupation would be placed over one of these scatters; however, this also creates a greater number of unheated lithics, so it is uncertain how this parameter will affect the overall percentage of heated lithics in a layer. Moreover, the proportion of lithics that are heated should remain the same regardless of whether the number of lithics per scatter are raised or lowered.
6. **Sediment as a thermal buffer**. Sediment functions as a thermal buffer by absorbing and dissipating heat [39, 41, 43]. Therefore, fire proxy percentages will decrease as the amount of sediment between occupation surfaces is increased. Higher volumes of interstitial sediment could be the result of increased sedimentation rates, longer temporal gaps between occupations, or a combination of both (see Section 2.3.).
7. **Fire-free occupations**. Introducing occupations without fires between those with hearth features will decrease the resultant number of heated artefacts within a layer. This is due in part to a) an effective reduction in the number of fire-bearing occupations within a layer, b) an increase in the amount of sediment between fire-occupations, creating an increased thermal buffer, and c) an increase in the lithic scatter-to-hearth ratio.
8. **Lithic artefacts introduced directly into the fire (hereafter, introduced lithics)**. Regardless of where lithic scatters are positioned in relation to a hearth during an occupation, only a small percentage of artefacts (perhaps around 1%, based on the findings at a number of probable single-occupation Palaeolithic sites [56–58]) will be accidentally introduced into the hearth (or on rare occasions, purposefully introduced [12, 59]), either while flintknapping, kicked in while walking by, or dropped in during other fireside tasks like cutting meat or repairing broken tools. We assume that this number is higher within more confined areas like small caves or constructed shelters, where limited space both decreases the distance between the hearth and flintknappers and increases foot traffic.

## 2. Methods and theory

### 2.1. The model: fiReproxies

We employ computer-based modelling in an attempt to better understand how the various parameters outlined within the hypotheses above can influence the production of fire proxy evidence. The model assumes that recurrent hominin occupation of a cave will generate lithic knapping scatters that are heated by fires subsequently placed inside the cave area, creating variable distributions of fire proxies depending on the chosen parameter values. The spatial extent of the model is stored in a grid in which the grid cells are either inside the cave, outside the cave or part of the cave wall. In a simulation, hearths are placed on the grid, heating any lithics that were previously deposited underneath. Hearths and lithics are distributed in layers throughout the cave in events referred to here as ‘occupations’. One or more occupations comprise a ‘layer’, which contains the results for a single set of modelling parameter values. In any one occupation, zero or more hearths are placed, and zero or more lithic scatters are deposited, their placement being either random or according to chosen preferences. Together, these number and placement preferences create different combinations of parameters that include occupation surfaces of different shapes or sizes, random fire placement (FR), fires placed near previous fires (FNP), uniform placement of lithics (LU), random placement of lithic (LR), random placement of discrete lithic scatters (LSR), lithic scatters placed near fires (LSNF), number of fires per occupation, number of lithic scatters per occupation, fire size and depth of heat penetration (i.e. the degree of thermal buffering [TB], see Section 2.3. below), the number of occupations without fires between occupations with hearths, and the number of occupations in total within a hypothetical archaeological layer (a quick reference list of the abbreviations discussed above is provided for the reader in S1 Table).

**Fig 1:**
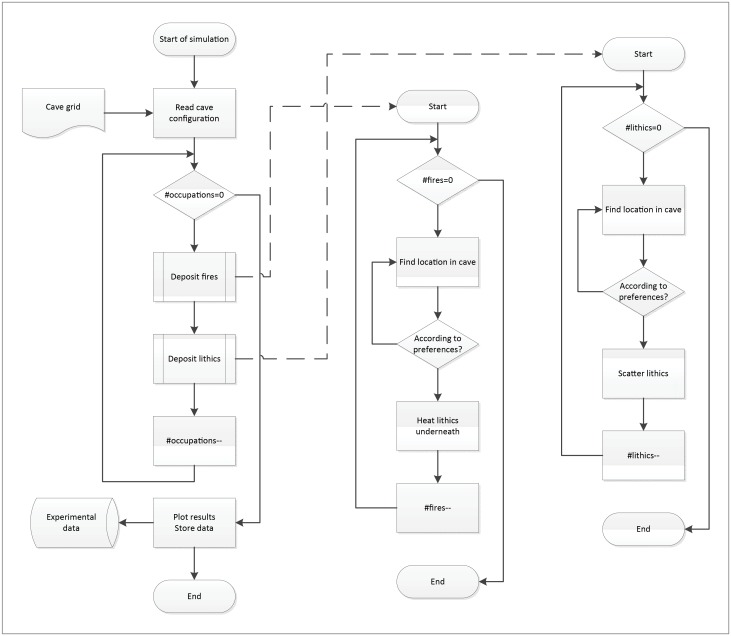
Flow diagram outlining the basic structure of the ‘fiReproxies’ computer simulation employed in this study.

**Fig 2:**
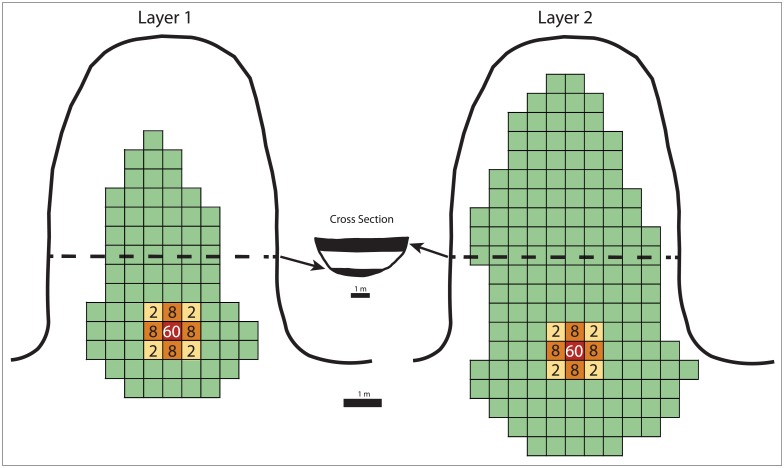
Diagrams depicting the layouts of the hypothetical cave and occupation layers utilized in the simulations. The grid cells delimit the extents of Layer 1 (left: 40 m^2^ in 80–50-cm^2^ units) and Layer 2 (right: 80 m^2^ or 160–50-cm^2^ units). The red, orange and yellow grid cells indicate the relative distribution of lithic fragments within individual lithic scatters, the numbers expressing the percentage of lithic artefacts deposited in each grid cell. Lithic artefacts are absent in green grid cells. The dashed lines delineate the stratigraphic cross-section of the cave deposits (center), with the shaded portions corresponding to Layer 1 (lower) and Layer 2 (upper).

In every occupation, all hearths are placed first, heating lithics scatters underneath immediately to the configured depth. Then lithics are scattered throughout the cave, thus avoiding heating from fires in the same occupation event. This process is depicted in the flow diagram in Fig 1. Each lithic scatter is deposited on the grid as shown in Fig 2c. For our experiments, 1% of the newly deposited lithics is assumed to be heated (i.e. accidentally introduced into a fire while burning), so 99% is deposited unheated (note: this parameter is also adjustable in the model to account for situations where more lithics might be introduced into a hearth). For administrative and statistical purposes, occupations are ordered per experiment, and a number of experiments are organized in sessions.

All simulation were run using R version 3.1.2 [69] and developed in RStudio version 0.98 [70]. Source code (S1 File) and a short user manual (S2 File) for the model are available in the supporting information.

### 2.2. The simulations

For a comprehensive list of the simulations performed and their selected parameters, please refer to S2 Table.

To determine how the size and shape of an occupation surface might influence the number of fire proxies produced, we first compared generic square-shaped and rectangle-shaped occupation surfaces varying sizes composed of 50-cm^2^ grid cells (squares: 4x4 to 25x25; rectangles: 3x6 to 18x36). Then, in an effort to demonstrate how our model might be utilized to help interpret archaeological fire proxy data, we apply our model to a hypothetical archaeological site (see Sections 2.5. and 4.2.).

Successive generations of contemporaneous hearths and flintknapping scatters (100 lithic fragments per scatter) are placed relative to one another within the layers. For this study, flintknapping scatters are assumed to be ca. 1 m-wide (cf. [71]), with the default setting of our model confining the majority of artefacts (60%) to the 50-cm^2^ centroid of a scatter, and placing 8% in adjacent grid cells and 2% in diagonal grid cells (Fig 2c). However, our model allows the user to vary the degree with which lithic artefacts are spread around the site, ranging from total homogenization (random or uniform distributions) to simulate post-depositional homogenization of lithics within cave deposits, to a scatter remaining entirely within the grid cell it was deposited. It is possible to implement other lithic scatter configurations within the model, but these were not tested here.

Hearths here are assumed to be between 50-cm- and 1-m-wide (cf. [18, 20, 72]). A 50-cm-wide hearth only affects extant artefacts within the grid cell it falls, while a 1-m-wide hearth affects the artefacts in four adjacent grid cells. Each series of hearth(s) and flintknapping scatter(s) comprises one occupation. In subsequent occupations, the sequential placement of hearths relative to previous flintknapping scatters is the primary mechanism that determines the number of lithic artefacts that are heated within a layer. Thirty (30) experiments are run for each set of selected parameter values, with the results of each session being the average and standard deviations of these experiments.

### 2.3. Thermal buffering

We introduce into our model the concept of *thermal buffering* (TB), which effectively reflects the percentage of underlying lithic artefacts any one fire will thermally alter within a grid cell. This parameter accounts for both the insulative effect of sediment and debris deposited between (and during) occupations, which can reflect geogenic (and anthropogenic) sedimentation rates and/or the site use frequency, as well as the intensity of a fire, with the heat generated by hotter, longer-burning fires generally penetrating more deeply into the substrate (cf. [39, 41]). For example, a low sedimentation rate coupled with a low return interval to the site in one archaeological layer could result in the same amount of sediment deposited between occupations as in a layer exhibiting a high sedimentation rate and a high return interval, potentially yielding comparable percentages of heated lithics. Within our model, very low TB (~0–5%) may correspond either to palimpsests, where there is virtually no sediment deposited between episodes of fire use that might inhibit heating, or to instances where high-temperature and/or long-duration fires with potentially more deeply penetrating heating fronts are employed that can overcome sediment barriers.

**Fig 3:**
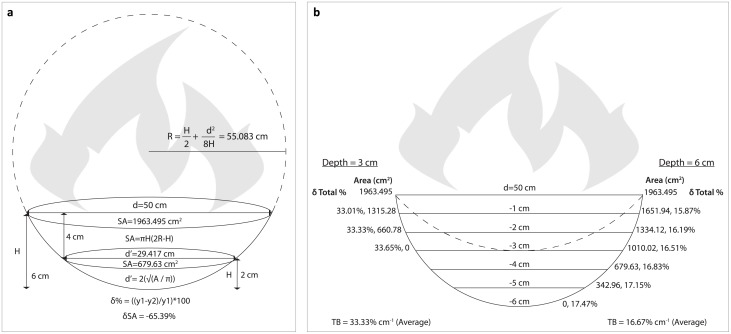
Diagrams showing the logic behind *thermal buffering* (TB) in our model. This is the rate at which extant lithic scatters are potentially heated in subsequent occupations. a) Assuming the 250°C isotherm of a 50-cm-wide hearth is able to penetrate 6 cm into the substrate, 100% of lithic fragments lying within the hearth’s footprint at the surface would be thermally altered, while <35% of the same lithic scatter would be thermally altered if buried 4 cm below the hearth due to the reduction in surface area exposed to the heat. b) The ‘δ Total %’ value per unit of depth change increases as the depth of penetration of the 250°C isotherm becomes more shallow and/or the unit of depth (corresponding in our model to the amount of sediment deposited between occupations) is increased. For simplicity, we use the average δ Total % value, rounded to the nearest whole value, as the TB value in our model. See S3 Table for TB value calculator based on these diagrams. (Note: Figures are not to scale. d = diameter, SA = surface area, H = height of the spherical cap).

Experimental hearths tend to create basin-shaped zones of reddened sediment ([39, 73]), resulting from the oxidization of trace amounts of iron (the limonite/goethite-to-hematite reaction series) at temperatures above ~250°C [74, 75]. According to Bessey [50], this colour change proceeds slowly at 250°C, requiring upwards of 18 hours to fully develop, whereas colours are fully developed after only 2 hours at 300°C. This 250–300°C isotherm is therefore the maximum depth at which one would expect underlying lithics to exhibit discoloration caused by heat alteration (cf. [39]). It should be mentioned, however, that overt evidence of excessive heating caused by thermal shock (fracturing, crazing, potlidding) likely only affects artefacts at or very near the surface underlying a fire [12, 43, 76]. The intensity of a hearth of known size can be roughly estimated based on the penetration depth of this isotherm, if present. Moreover, by assuming the shape of the basin equates to a spherical cap, we can quantify not only the volume of the heated sediments, but also the horizontal surface area heated at a particular depth, giving us the ability to test variable TB values based on differences in hearth size and heat penetration depth (Fig 3).

Using the information outlined in Fig 3 and S3 Table, one can calculate TB for different scenarios regarding both fire intensities and the amount of sediment between occupations. For example, a 50-cm-wide hearth covers an area of 1963.5 cm^2^, meaning 100% of the lithics within this zone at the surface would theoretically be heated (i.e. 0% TB). If this same lithic scatter is located 4 cm below the surface, and this hearth exhibits a 6-cm-deep reddened basin, then it is only possible to heat around 35% of the lithics (relative to the surface scatter), since only 679.63 cm^2^ of the occupation surface directly underlying the hearth can be heated at that depth (Fig 3a). The depth to which the heat penetrates dictates the shape of the rubefied basin, with more deeply penetrating basins having steeper sides (assuming similar hearth size). In our model, the depth of the heated basin, when coupled with the occupation rate (e.g. one occupation for every 1 cm or 0.5 cm of sedimentation, etc.), dictates the rate of TB for a layer based on the total percent change per occupation for a known heating depth (Fig 3b). This value varies slightly with depth, but for the sake of simplicity, we use the average of these values (rounded to the nearest whole number) as the TB value for the layer being simulated. Returning to our hypothetical hearth, TB for a 50-cm-wide hearth with a 6 cm deep heated zone increases with depth by around 17% per cm (average value), meaning an occupation rate of one occupation every 2 cm would have a TB value of 33% (average value). The same hearth with a 3-cm-deep heated zone also increases TB to around 33% per cm (Fig 3b).

### 2.4. Model considerations

The old adage stating "All models are wrong (but some are useful)" [77] also applies to ours, as it is built on numerous assumptions, the most obvious being that conditions (e.g. the number of fires, the number of lithic scatters, the TB) remain consistent throughout a modelled layer. Variability is the rule in human activity, so to assume any sort of rigid continuity between occupations is of course unrealistic. However, in order to determine how different parameters within our model influence the production of fire proxies, some control in the form of variable consistency is essential. Nevertheless, some conditions can be safely estimated (e.g. occupation surface size) and are at times moderately consistent (e.g. climatic conditions), at least for the temporally constrained periods of time encompassed within one layer.

The purpose of our model, therefore, is not to create a perfect imitations of realistic occupation processes, but to test the proposed parameters, evaluate their respective sensitivities and provide predictions of how different behavioural scenarios will manifest in the archaeological record. It is thus impossible to account for all the variables influencing how and when fire was use by prehistoric peoples, including rational (and sometimes irrational) choices made by human groups regarding campsite structuring based on environmental or site-specific conditions encountered in a cave (e.g. issues of space, ground conditions or airflow within the cave, exterior weather conditions, etc.). Some insight into these choices may be gained in the future by conducting more actualistic experiments within a cave or some other confined space or comparing our finding with those encountered in the ethnographic record. Also, following [9], we do not factor into our model the potential for increased percentages of heated artefacts through thermal fracturing. The aforementioned study has shown that including only flakes retaining their proximal ends (i.e. striking platforms) in the artefact sample used to calculate percentages of heated elements yields comparable relative trends between layers as does including all lithic fragments (broken and unbroken) in the sample, even though the latter percentages tend to be higher overall.

### 2.5. Model exploration

While our model could theoretically be applied to any archaeological site possessing evidence for fire use, as a practical exercise, we applied our model to a generic Middle Palaeolithic cave site wherein we compared two hypothetical archaeological layers, each representative of disparate climatic periods and depositional settings. We rely on known archaeological and palaeoclimatological data for western Europe during Last Glacial period to reconstruct the values for various parameters influencing the production of heated lithics within these layers [10, 78–86]. The lower Layer 1 is the smaller of the two layers, with a thickness of 7.5 cm and comprising an occupational surface area of 20 m2 (80 grid cells), and is meant to simulate a layer deposited early in the use life of the cave during warmer interstadial conditions (Fig 2a). The upper Layer 2 encompasses twice the surface area of Layer 1 at 40 m2 (160 grid cells) and a greater thickness of 45 cm, reflecting how the sedimentological infilling of the cave increased during this period, creating a larger habitable surface (Fig 2b). For the purposes of our exercise, Layer 2 simulates a layer deposited during a colder stadial period. Each layer is occupied 30 times at regular intervals, with the thicknesses of the layers reflecting their respective sedimentation rates: 0.25 cm between occupations for Layer 1, and 1.5 cm for Layer 2. The percentages of heated lithics for each layer are modelled based on the values selected for each parameter discussed above in Section 1.1. (see also the fiReproxies User Manual, S2 File). How and why we set the values for the model parameters as we do are discussed later in Section 4.2.

With this hypothetical case study, we aim to 1) test the effects the various parameters have on the generation of heated lithic artefacts, and 2) demonstrate how our model can be used as a tool to determine what combination(s) of conditions could result in fire proxy percentages observed at actual archaeological sites.

## 3. Results

Here we outline the results of our simulations in a format relatable to our initial hypotheses in Section 1.1.

**Fig 4:**
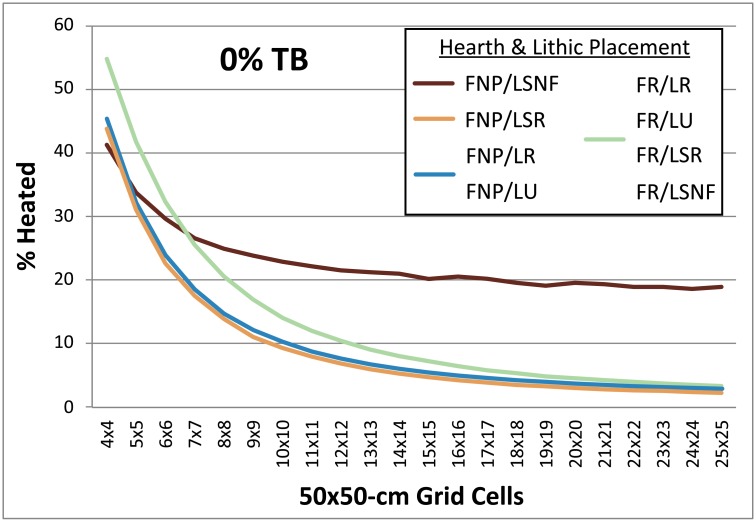
Chart comparing occupation surface size and various fire and lithic scatter placement scenarios. Parameters: 1 fire, fire size 1, 4 lithic scatters, 30 occupations, 1% introduced lithics. Given the near identical results obtained for simulations using the FNP/LR and FNP/LU settings (blue), as well as for all simulations using the FR setting (green), for the sake of clarity these are shown here as single curves. FNP = Fire near previous, FR = Fires random, LSNF = Lithic scatters near fire, LSR = Lithics scatters random, LR = Lithics random, LU = Lithics uniform, TB = Thermal buffering.

**Fig 5:**
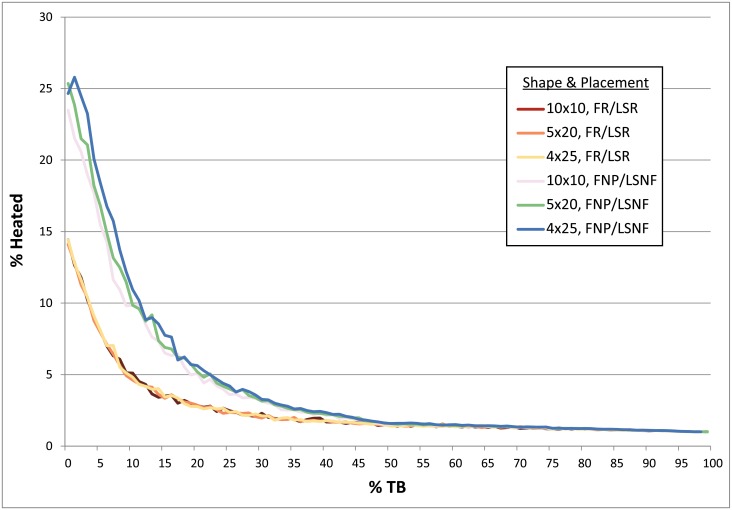
Chart comparing same-sized occupation surfaces of different shapes (i.e. degree of elongation) and different hearth and lithic placement scenarios. Parameters: 1 fire, fire size 1, 4 lithic scatters, 30 occupations, 0–100% TB, 1% introduced lithics. FNP = Fire near previous, FR = Fires random, LSNF = Lithic scatters near fire, LSR = Lithics scatters random, TB = Thermal buffering.

**Fig 6:**
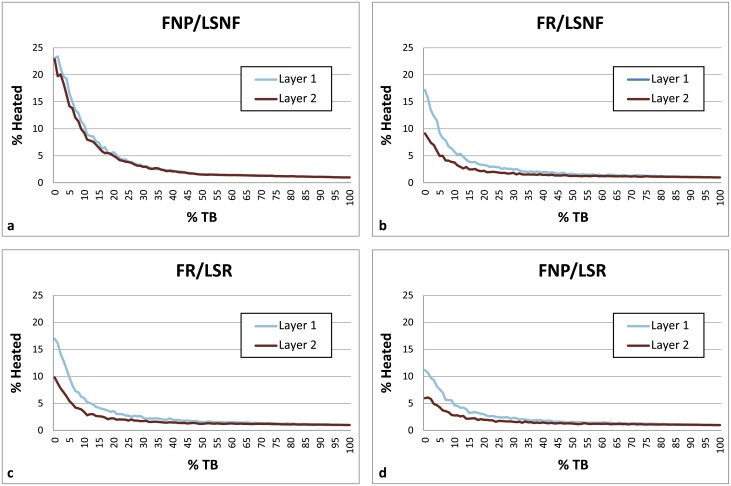
Charts comparing fire and lithic scatter placement scenarios between Layer 1 and Layer 2. Parameters: 1 fire, fire size 1, 4 lithic scatters, 30 occupations, 0–100% TB, 1% introduced lithics. The heated lithic percentages and curves for the FR/LR and FR/LU settings are virtually identical to those seen in the FR/LSNF and FR/SLR charts, while the plots using the FNP/LR and FNP/LU settings are nearly identical to those seen in the FNP/LSR chart, so these were not included here but were included in S1 Fig in the Supporting Information. FNP = Fire near previous, FR = Fires random, LSNF = Lithic scatters near fire, LSR = Lithic scatters random, LR = Lithics random, LU = Lithics uniform, TB = Thermal buffering.

**Fig 7:**
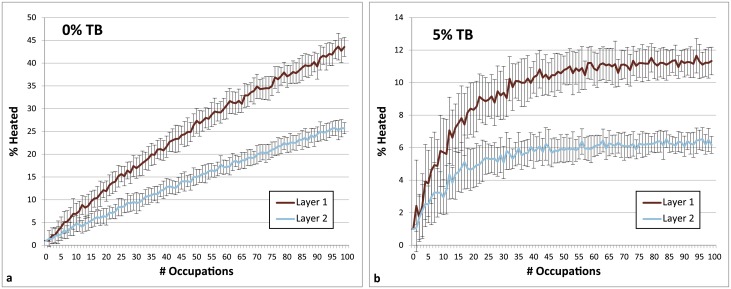
Charts demonstrating how the number of occupations per layer impacts heated lithic percentages for Layer 1 and Layer 2. Parameters: Fires Random/Lithic Scatters Random (FR/LSR), 1 fire, fire size 1. 4 lithic scatters, 0% and 5% TB, 1% introduced lithics.

**Fig 8:**
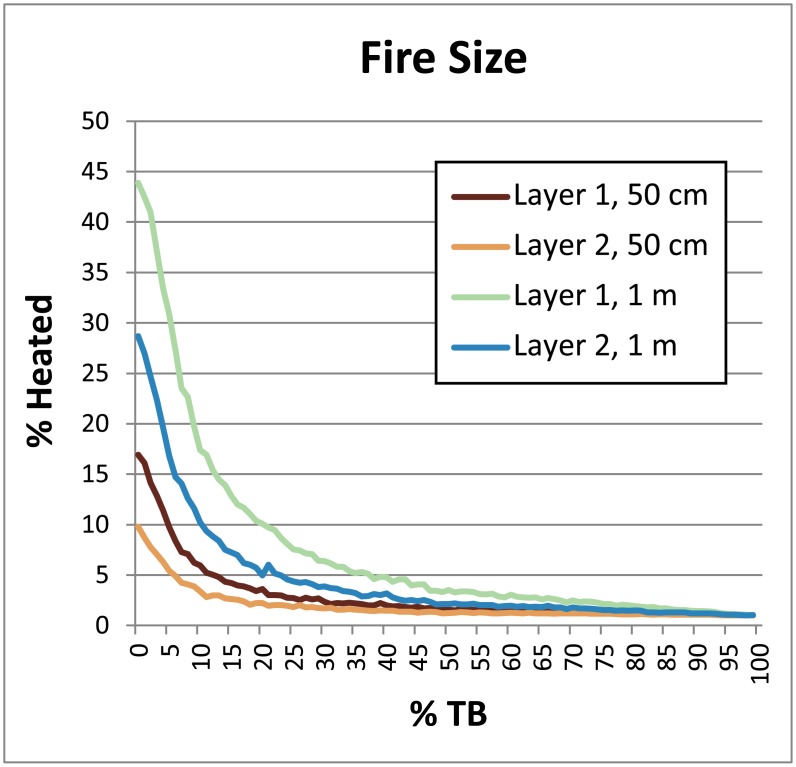
Chart demonstrating the effects of fire size on the percentage of heated lithics. Parameters: Fires Random/Lithic Scatters Random (FR/LSR), 1 fire, 4 lithic scatters, 0–100% TB, 1% introduced lithics. While a 1 m-wide fire (fire size 4) is effectively a cluster of four 50 cm-wide hearths (fire size 1) in our model, note the slightly lower relative percentages compared to four individually placed 50 cm-wide hearths in Fig 9.

**Fig 9:**
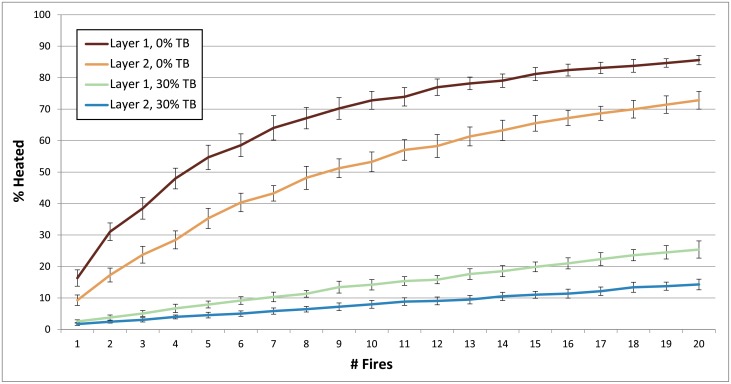
Chart demonstrating the effects of increasing the number of fires per occupation on fire proxy production for Layer 1 and Layer 2. Parameters: Fires Random/Lithic Scatters Random (FR/LSR), fire size 1, 4 lithic scatters, 30 occupations, 0% and 30% TB, 1% introduced lithics.

**Fig 10:**
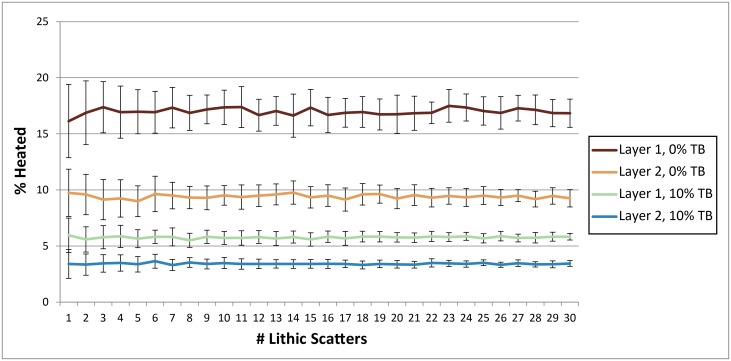
Chart showing how the number of lithic scatters per occupation influences heated lithic percentages for Layer 1 and Layer 2. Parameters: Fires Random/Lithic Scatters Random (FR/LSR), 1 fire, fire size 1, 30 occupations, 0% and 10% TB, 1% introduced lithics.

**Fig 11:**
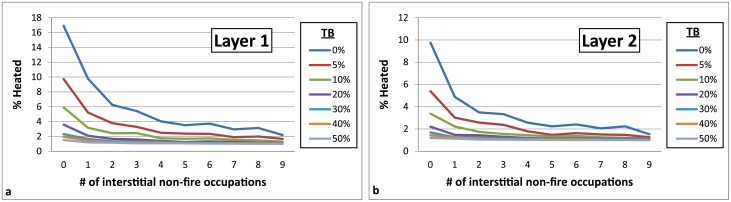
Chart demonstrating the effects of placing occupation layers without fires between fire-bearing occupation layers. Parameters: 30 total occupations, Fires Random/Lithic Scatters Random (FR/LSR), 1 fire, fire size 1, 4 lithic scatters, 0–100% TB, 1% introduced lithics.

**Fig 12:**
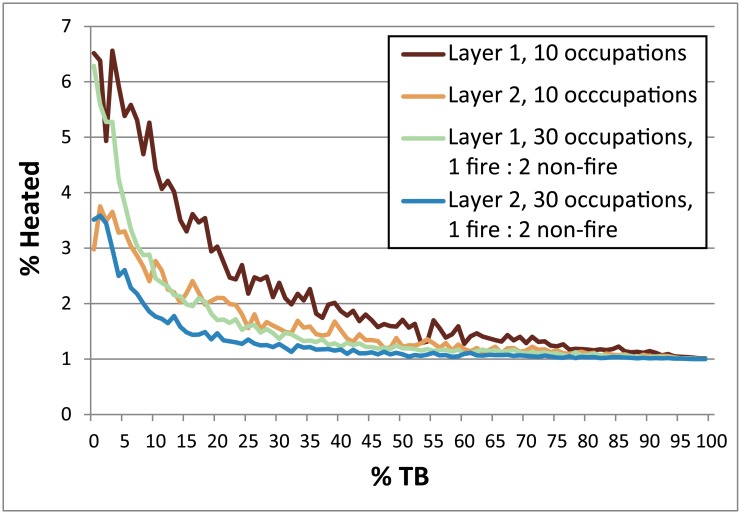
Chart comparing a layer with hearths every third occupation (30 occupations total) with a layer with 10 occupations and fire in every occupation. Parameters: Fires Random/Lithic Scatters Random (FR/LSR), 1 fire, fire size 1, 4 lithic scatters, 0–100% TB, 1% introduced lithics.

**Fig 13:**
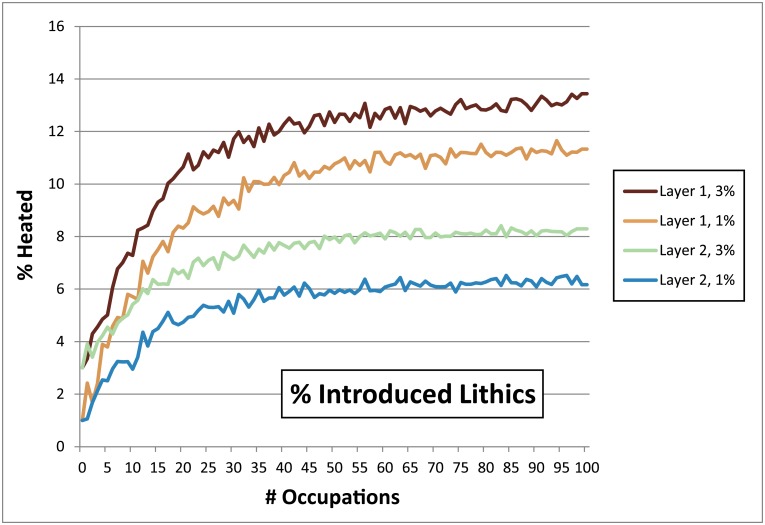
Chart demonstrating the effects of increasing the number of lithic artefacts introduced directly into a hearth (i.e. introduced lithics, see Section 2.1) from 1% to 3%. Parameters: Fires Random/Lithic Scatters Random (FR/LSR), 1 fire, fire size 1, 4 lithic scatters, 5% TB. Changing this percentage only appears to affect the final percentages by around the same number.

1. **Occupation surface size and shape**. Smaller occupation surfaces consistently yield higher percentages of heated lithics than larger surfaces, all other parameters being the same (Fig 4). Occupation surface shape does not appear to influence fire proxy production when fires are randomly placed (FR) with discrete lithic scatters also placed randomly (LSR) (Fig 5). However, when fires are placed near previous fires (FNP) and lithic scatters placed near fires (LSNF), then the more elongated shapes yield slightly higher percentages of heated lithics (despite identical surface areas), most notably below 30% TB. This is likely due to the constriction of the site area limiting where subsequent hearths can be placed, thus increasing the potential for overlap.
2. **Relative placements of hearths and lithic scatters**. Simulations using random and uniform distributions of lithic artefacts (LR and LU) produce results that are virtually identical to those using randomly placed discrete lithic scatters (LSR) (Fig 4). Using the LSR setting, where lithic scatters bleed into adjacent grid cells, it is possible to completely blanket an occupation surface with lithics after depositing as few as 10–20 lithic scatters, depending on the size of the occupation surface (i.e. after only ~ 2–5 occupations, assuming four scatters are laid down per occupation). Thus, it is likely that after a number of occupations, especially in palimpsest situations, the superimposition of many multiple lithic scatters will give the impression of a nearly homogeneous distribution. Simulations where fires are placed near previous fires (FNP) and the lithic scatters placed near fires (LSNF) yield the highest percentages overall, except in very small areas (Fig 4). In random lithic placement scenarios (LSR, LR, LU), percentages are higher when the fire placement is also random (FR), as opposed to FNP (Figs 4 and 6). In our case study, Layer 1 and Layer 2 percentages are most similar in FNP/LSNF scenarios, all other parameters being the same. This configuration appears to reduce the effective size of the site for both layers, making them comparable (Fig 6a).
3. **Number of occupations per layer**. The percentage of heated lithics increases consistently as the number of occupations increase, assuming 0% TB (Fig 7a). However, as TB is increased, the percentage of heated lithics produced per occupation will plateau after a point, with the plateau beginning sooner at higher rates of TB (e.g. at 5% TB, the curve plateaus after around twenty occupations [Fig 7b], at 10% TB a plateau can be seen around ten occupations [Simulation Log 21], and at 20% TB around five [Simulation Log 22]).
4. **Nature and number of fires**. Archaeological hearth features are generally 50–100 cm wide, and our simulations suggest that increasing the size of the fire in an occupation from the former to the latter dimensions can potentially double to more than triple the number of fire proxies produced (Fig 8). Increasing the number of fires burning per occupation consistently yields higher percentages of heated lithics (Fig 9).
5. **Number of lithics per occupation**. Increasing the number of lithic scatters per occupation, however, only appears to reduce the standard deviation of the mean percentages recorded, while the mean percentages themselves remain largely consistent (Fig 10).
6. **Sediment as a thermal buffer**. As is demonstrated in most of the graphs presented here, any increase in thermal buffering—whether caused by thicker sediment packaged between occupations, or by deeper penetration of heat caused by increased fire intensity (temperature and/or duration)—decreases the resultant percentage of heated lithics (Figs 5, 6 and 8–13).
7. **Fire-free occupations**. Inserting fire-free occupations between fire-bearing occupations reduces the resultant percentages of fire proxies (Fig 11). Fig 12 demonstrates how the fire-free occupations not only reduce the heated lithic percentages by reducing the number of fire occupations in a layer, but that the increased amount of sediment between fire-layers also effectively increases TB.
8. **Lithic artefacts introduced directly into the fire (i.e. introduced lithics)**. Increasing the percentage of lithic fragments introduced into hearths during an occupation only increases the final percentages by roughly this percentage (i.e. if 1% or 3% of lithics are introduced into the fire, the final percentage is approximately 1% or 3% higher) (Fig 13). This trend holds for the different hearth or lithic placement configurations, as well as for different TB.

## 4. Discussion

### 4.1. General discussion of modelling results

It is clear that some of the modelled parameters are more influential than others at affecting the rates at which heated lithic artefacts are produced. Obviously, when fire is used very infrequently, only low amounts of heated lithics are produced (Fig 11). What is interesting, however, is that similarly low percentages of fire proxies can also be produced in simulations where fire is omnipresent, depending on the parameter values used. A layer that is occupied once or perhaps only a handful of times tends to have lower percentages than a layer containing many occupations (Figs 7 and 13). Except in a pure palimpsest situation (TB = 0%), this increase in the percentage of heated lithics is more significant between 'a few' and 'some' occupations (<5 versus 20) than between 'some' and 'a lot' (20 versus 100).

The degree of thermal buffering provided by the amount of sediment that is deposited between occupations is also very important. The difference between a palimpsest situation with virtually no sediment being deposited between occupations (<5% TB) and one where comparatively thick sediment packages lie between occupations (>50% TB) can result in relatively high percentages for the former, and virtually no heated lithics in the latter (Figs 5 and 6). Fire proxy percentages are also dependent both on hearth and lithic scatter placement settings, with the FNP/LSNF setting yielding the highest values (Figs 4–6), and on the size of the occupation surface (Figs 4 and 6). This latter parameter appears to be more important for very small sites than larger sites, where, for example in Fig 4 (FR/LSR), the total change between a rough doubling of the surface area is greater between a surface comprised of 49 (7x7) and 100 (10x10) grid cells at 12.29% (26.01% versus 13.72%, respectively). The difference between 196 (14x14) and 400 (20x20) grid cells is only 3.12% (7.69% versus 4.57%, respectively). Considering these values, comparing two archaeological sites with vastly different surface areas (all other factors being equal) can have major implications for how researchers might (mis)interpret the relative difference in the degree of fire use between these areas.

Increasing the number of fires or the fire size produces similar effects in the model, with both, as expected, yielding higher numbers of fire proxies (Figs 8 and 9). However, both are subject to real world practicalities, like fuel availability, spatial restrictions, or even the need for very large or many fires in multiple localities within an occupation. This will likely limit the number of fires burning on a site at any one time to a handful, while confined fires of very large proportions would be limited to very infrequent, perhaps more specialized purposes. However, purposeful surface fires may have occasionally been lit in caves to clear away old grass bedding and the pests living therein [87, 88]. The effects that such fires would have on artefacts lying at or near the surface would largely depending on the thickness and degree of compaction of the accumulated fuel [89].

Increasing the number of lithics introduced onto a site during an occupation will increase the *absolute amounts* of heated lithics within a layer, but this has little effect on the final *percentage* of heated lithic produced, so long as fire use is consistent between occupations (Fig 10). This parameter becomes important only when additional lithics are added during fire-free occupations (Fig 11). This makes the total number of lithics recovered from an archaeological layer a poor guide for understanding the frequencies of heated lithics within a deposit, though the total number of lithics and the number of lithic types produced (e.g. cores, tools) could potentially be helpful in estimating the number of occupations (both relatively between layers, and perhaps absolutely within a layer).

The introduction of a small amount of lithic material into a burning fire appears to be the norm on sites where stone tools were produced and used. We assumed this to be a low number per occupation (perhaps around 1%) as inferred from the relatively low number of heated artefacts produced at larger open-air (probable) single-occupation sites (cf. [56–58]), though this percentage could potentially be higher (possibly as high as 5%) within the confines of a very small cave or a constructed shelter (perhaps <20 m^2^ surface area) (cf. [47, 90]). These numbers, however, do not take into account the possibility that at times lithic raw materials may have been purposefully introduced into hearths (e.g. [12, 59]), though given that the thermal shock caused by rapid heating of fine-grained siliceous stone potentially has explosive consequences [43, 49], the reasons for doing this are unclear. Nevertheless, for whatever reason, adding large quantities of lithic debris to a fire would drastically increase the overall percentages of heated lithics in any deposit [12].

### 4.2. Applying fiReproxies model to a hypothetical case study

**Fig 14:**
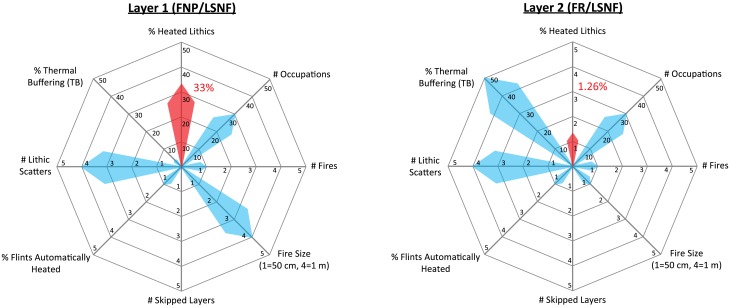
Rose plots providing visual representations of the Layer 1 and Layer 2 occupation scenarios described in section 4.2. The red arrows and associated red numbers indicate the resultant percentages of heated lithics produced in each scenario, while the values of other parameters are depicted as blue arrows. Note the different scales for heated lithics between Layer 1 and Layer 2. FNP = Fire near previous, FR = Fires random, LSNF = Lithic scatters near fire.

In this section, we demonstrate the utility of our model by applying it to a hypothetical Middle Palaeolithic cave site, as outlined in Section 2.5. Visual representations of the parameters selected and the resultant percentages of heated lithics produced in each of the hypothetical scenarios described below for Layers 1 and 2 can be seen in Fig 14.

#### Scenario for Layer 1

Layer 1 was deposited early in the use life of the cave, with the shape of the cave allotting only 20 m^2^ of floor space to the inhabitants (Fig 2a). Prevailing warm climatic conditions fostered a forested environment wherein the occupants hunted local, non-migratory prey species allowing for lower residential mobility (i.e. more frequent and longer periods of use of the cave). Geogenic sedimentation rates were low (e.g. 0.25 cm between each occupation) allowing subsequent occupants to observe approximately where the previous hearth was placed, leading them to preferentially place their hearth within the diffused ash scatter of the previous hearth (FNP). Flintknapping generally took place nearby the hearth. Trees grew in abundance in the vicinity of the cave providing easy access to fuel which, largely out of convenience, allowed for larger, hotter fires burning for longer periods of time with minimal extra effort. The 250°C isotherm generally had ample time and energy to achieve a depth of 6 cm below the surface. Under such conditions, we could expect around 19.28 ± 4.9% of the lithic debris bearing evidence of heating after 30 visits to the cave at 4% TB (this value based on the low sedimentation rate and depth of heat penetration of the fires; see Fig 3 and S3 Table). If fuel and the need were sufficient, having two 50-cm-wide fires burning simultaneously would have increased this amount to 24.22 ± 5.13%, while employing instead a larger 1 m-wide fire per occupation would potentially raise the heated lithic percentage for the layer up to 33.01 ± 6.82% (Fig 14). This number could potentially be higher still (perhaps by one or two percent) if one assumes the more confined space would allow for increased numbers of lithics to be incidentally introduced into the fire while it is burning.

#### Scenario for Layer 2

By the time Layer 2 is deposited, infilling of the cave has doubled the available floor space to 40 m^2^ (Fig 2b), which according to our model would reduce fire proxies to 28.19 ± 6.78% from Layer 1 levels, also assuming 30 total occupations for this layer. Meanwhile, climatic conditions have become much colder. The local red deer have become sparse, while migratory reindeer have become the preferred prey species, increasing group mobility and ultimately reducing site use frequency and duration. An increase in sedimentation rates caused by increased freeze-thaw action, coupled with the reduced site use frequencies, has increased the thickness of the sediment packages between occupations to 1.5 cm. Since previous hearth positions are likely invisible to subsequent inhabitants, this visual cue to place a hearth in a similar place is perhaps negated, creating more or less random hearth placement (FR), again lowing the percentages to 21.25 ± 2.77%. Woodlands have largely given away to steppic landscapes, where mostly grasses, shrubs and the occasional small stand of dwarfed trees are located in the vicinity of the cave. The reduction in available fuel allowed for only one 50-cm-wide fire at any one time, which would have reduced the percentage of fire proxies to 5.94 ± 1.0%. To conserve fuel, fires burned with lower intensities and for shorter durations, thus reducing the depth of heat penetration into the substrate to 3 cm for the 250°C isotherm. Together with the increased sedimentation rate, these variables effectively increase TB to 50%, lowering the total percentage of fire proxies for the layer to a meagre 1.26 ± 0.18% (Fig 14).

These cold and warm scenarios are just two of numerous possible configurations that could yield similar results. For example, had Neandertals in the colder period only sporadically used fire (cf. [10]), say only every sixth occupation (skip 5), then assuming all other parameters stayed the same between Layer 1 and Layer 2 (minus the hearth placement parameter necessarily changing from FNP to FR, since the additional sediment between the layers would obscure previous hearths), then the percentage of fire proxies produced in Layer 2 already drops significantly to 3.72 ± 1.10%. Fewer visits to the site in general would also lower percentages (Fig 7). Altering some parameters could also in effect cancel one another out; for example, an increase in site use frequency could compensate for an increase in sedimentation rates, or an increase in the total number of occupations could compensate for higher TB. In the end, our model does not provide at this stage a concrete explanation of events at a prehistoric site, but instead allows users to test which configurations of parameters, inferred from the archaeological realities for a particular site or layer, might yield observed fire proxy percentages. This is particularly interesting for layers with high levels of fire proxies, which tend to be more exceptional and seem require special circumstances to achieve.

### 4.3. Examples of archaeological applications

Primary fire residues like charcoal and ashes are often removed from archaeological substrates via various diagenetic or taphonomic processes. Likewise, other indications of burning like rubefied sediments underlying hearths may be only weakly developed or absent entirely, as can sometimes be the case in sandy soils [91] or in karstic sediments derived from limestone [20, 39]. Potentially understanding the conditions behind fire proxy production is important in such difficult situations where associated fire indicators are lacking.

There are numerous instances where fire proxy evidence is present within archaeological layers at Middle Palaeolithic sites that possess little to no other evidences of on-site burning (consult Dataset S1 in [92] for examples and their associated sources). This is the case, for instance, in the upper Layers 4–2 at the French Middle Palaeolithic site of Roc de Marsal (Dordogne), wherein fire use is alluded to by the presence of low percentages of fire proxies, but no primary combustion features or their residues have been observed ([10, 17, 18]). This is in stark contrast to the lower Layers 9–5—deposited during a warmer climatic period—that possess higher percentages of fire proxies and numerous intact combustion features (Layer 9 and 7). This evidence is interpreted by the authors as a clear-cut indication for less frequent fire use in the upper layers likely related to the prevailing cold climatic conditions during this period reducing Neandertal access to natural fires, thus suggesting they could not make fire themselves ([10, 93, 94]; but see [62]). While this could very well be the case, this scenario being one supported by our model when multiple fire-free occupations are introduced between occupations where fire is used (see previous section, Figs 11 and 12), the scenario outlined in Section 4.2. could also potentially explain the pattern at Roc de Marsal. In this scenario and at Roc de Marsal, the percentages of heated lithics within the layers correlate closely to their respective lithic artefact densities throughout the sequence (see Fig 7 in [10]). Higher percentages of heated lithics (ca. 30–6%) occur in the layers with higher artefact densities (ca. 5–2.8 artefacts per litre of sediment in Layers 9–5, respectively), perhaps reflecting a palimpsest effect caused by lower geogenic sedimentation rates and/or higher occupation frequency/intensity at the site during the warmer climatic interval. Conversely, layers with lower percentages of heated lithics (ca. 1.3–2% in Layers 4–2, respectively) also have lower artefact densities (ca. 0.9–0.4 artefacts per litre of sediment), possibly reflecting a higher rate of sedimentation and/or reduced site use frequency and/or intensity during the colder climatic period.

In contrast to the Roc de Marsal case, the model could also potentially be used to explain why in some contexts primary evidence for burning on-site is abundant, but concentrations of heated lithics are lower than would be expected. This could, for example, apply to the Early Middle Palaeolithic Layers J68–J63 (Unit IX, Tabun D) at Tabun Cave in Israel, where the proportion of heated lithics is smaller than in the preceding and following layers despite exhibiting clear evidence of fire use [9]. If fire use was already habitual by this point among the Levantine Neandertals, as is argued by Shimelmitz and colleagues, then other factors must account for these lower numbers. As our model suggests, reduced site use frequencies coupled with higher sedimentation rates could result in such lower numbers, despite conditions being favourable for the preservation of primary fire residues. This would also explain the relatively low numbers of artefacts recovered from these layers and low rates of recycling in Unit IX [95]. Climatic deterioration may account for this shift, as well as reduced sea levels placing the Mediterranean shoreline farther from the cave [96].

While this study focuses specifically on heated lithic debris, the model could easily be adapted to fire-altered bone, as well, based on experimental data that has shown that high rates of post-depositional fragmentation can affect bones introduced directly into fires, while bones from previous occupations underlying a hearth will most likely only be charred and preserved as larger fragments [42]. This has implications both for sampling, where larger fragments are selected for analysis that exceed the average size of the resultant fragments, thus causing heated elements to be underrepresented [14, 15, 40], as well as for depositional settings, where, again, thicker sediment packages between occupations and/or low intensity burning can potentially leave underlying bones unaffected or only weakly affected.

## 5. Conclusions

Reproducing the conditions necessary to account for varying levels of fire remains in the archaeological record is exceptionally difficult. The interplay between the large number of possible variables (known and unknown) is often too complex to allow for a precise calculation of expected fire proxies in any given context. Nevertheless, we have made a first effort to do so with our computer-based fiRproxies model, which attempts to quantitatively assess the relative influence of some of the more important factors affecting how fire impacts lithic artefacts contained in underlying archaeological deposits. By modelling the effects of occupation surface area, the number of occupations, the amount of sediment deposited between occupations, the size and intensity of hearths, and different hearth and lithic scatter placement schemes, we have shown how altering one or more of these variables can have a considerable effect on the number and distribution of fire proxies produced within a modelled archaeological layer—even in scenarios where fire is consistently employed during every occupation. Our model moves beyond the simplistic view that ‘more fire means more heated lithic artefacts’ by introducing a means to assess how different archaeologically assessable environmental and behavioural variables, as defined by the aforementioned parameters, can be combined with known fire proxy data from an archaeological layer/site to arrive at a limited number of plausible fire use scenarios, wherein the relative impact of each parameter can be gauged. It is our hope that this model, in concert with other fire-related analyses and fire proxy data, will provide fundamental insights into possible site-specific conditions that are necessary to achieve known percentages of fire proxies in archaeological deposits, thus helping researchers better understand how and when fire was used by prehistoric peoples.

## Supporting information

S1 Fig Expanded version of Fig 6 displaying the charts comparing fire and lithic scatter placement scenarios between Layer 1 and Layer 2. Parameters: 1 fire, fire size 1, 4 lithic scatters, 30 occupations, 0–100% TB, 1% introduced lithics. FNP = Fire near previous, FR = Fires random, LSNF = Lithic scatters near fire, LSR = Lithic scatters random, LR = Lithics random, LU = Lithics uniform, TB = Thermal buffering.
(EPS)

S1 Table Quick reference list of abbreviations used in the text. (DOCX)

S2 Table List of simulation logs contained in S3 File. The parameters used for each simulation are defined, as are the figures and values mentioned in the text to which each simulation relates.
(XLSX)

S3 Table Thermal buffering (TB) value calculator based on known hearth widths and heat penetration depths. See Fig 3 for a visual representation of how the values obtained are used to calculate TB.
(XLSX)

S1 File fiReproxies source code, version 1.02. (R)

S2 File fiReproxies user manual, version 1.02. (DOCX)

S3 File Compressed folder containing simulations logs. Simulation logs 1–43 (See S2 Table).
(ZIP)

S4 File Compressed folder containing the area files used in the simulations. Layer 1 = Cave Small, Layer 2 = Cave Large, Squares 4x4 to 25x25, Rectangles 2x4 to 18x36.
(ZIP)
