# Supplementary material for: fiReproxies: A computational model providing insight into heat-affected archaeological lithic assemblages
Source: PLoS One. 2018 May 16;13(5):e0196777. doi: 10.1371/journal.pone.0196777 (PMC5955532; doi:10.1371/journal.pone.0196777)
Supplement: S1 Table — (DOCX) [file pone.0196777.s002.docx]

| Terminology | Short Terminology | Abbreviation |
| --- | --- | --- |
| Fires placed randomly | Fires Random | FR |
| Fire placed near previous fires | Fires Near Previous | FNP |
| Uniform placement of lithics | Lithics Uniform | LU |
| Random placement of lithics | Lithics Random | LR |
| Random placement of discrete lithic scatters | Lithic Scatters Random | LSR |
| Lithic scatters placed near fires | Lithic Scatters Near Fire | LSNF |
| Thermal buffering |  | TB |

S1 Table. Quick reference list of abbreviations used in the text.
