# Supplementary material for: fiReproxies: A computational model providing insight into heat-affected archaeological lithic assemblages
Source: PLoS One. 2018 May 16;13(5):e0196777. doi: 10.1371/journal.pone.0196777 (PMC5955532; doi:10.1371/journal.pone.0196777)
Supplement: S2 File — (DOCX) [file pone.0196777.s006.docx]

fiReproxies

User Manual

version 1.02

Fulco Scherjon and Andrew C. Sorensen

Human Origins group, Faculty of Archaeology

Leiden University, The Netherlands

Contents

[Introduction 2](#_Toc511643148)

[Installing the software 3](#_Toc511643149)

[Setup of the system 3](#_Toc511643150)

[Layout of the cave area files 4](#_Toc511643151)

[Running simulations 4](#_Toc511643152)

[Output of the simulations 5](#_Toc511643153)

[Changing the model 6](#_Toc511643154)

[Model parameters 7](#_Toc511643155)

[Simulation variables 8](#_Toc511643156)

[References 9](#_Toc511643157)

# Introduction

This model was developed to better understand how various parameters influence the production of fire proxy evidence. The model assumes that recurrent hominin occupation of a cave will generate lithic knapping scatters that are heated by fires subsequently placed inside the cave area, creating variable distributions of fire proxies depending on the chosen parameter values. The spatial extent of the model is stored in a grid in which the grid cells are either inside the cave, outside the cave or part of the cave wall. In a simulation, hearths are placed on the grid, heating any lithics that were previously deposited underneath. Hearths and lithics are distributed in layers throughout the cave in events referred to here as ‘occupations’. One or more occupations comprise a ‘layer’, which contains the results for a single set of modelling parameter values. In any one occupation, zero or more hearths are placed, and zero or more lithic scatters are deposited, their placement being either random or according to chosen preferences. Together, these number and placement preferences create different combinations of parameters that include occupation surfaces of different shapes or sizes, random fire placement (FR), fires placed near previous fires (FNP), random placement of discrete lithic scatters (LSR), lithic scatters placed near fires (LSNF), random placement of lithics (LR), uniform placement of lithics (LU), number of fires per occupation, number of lithic scatters per occupation, fire size and depth of heat penetration (i.e., the degree of thermal buffering), the number of occupations without fires between occupations with hearths, and the number of occupations in total within a hypothetical archaeological layer

In every occupation, all hearths are placed first, heating lithics scatters underneath immediately to the configured depth. Then lithics are scattered throughout the cave, thus avoiding heating from fires in the same occupation event. A percentage of the newly deposited lithics may be assumed to be heated (i.e., accidentally introduced into a fire while it is burning) with the remainder deposited unheated. Occupations are ordered per experiment, and a number of experiments are organized in sessions. All simulation are run using R version 3.1.2 ([R Core Team, 2014](#_ENREF_65)) and developed in RStudio version 0.98 ([RStudio Team, 2015](#_ENREF_69)).

This manual describes how to install the software and setup the system, how to run simulations, and how and where to change any model parameter values.

# Installing the software

The fiReproxies system is comprised of the following files, delivered in one zip file:

| Filename | Description |
| --- | --- |
| fiReproxies.R | The main source file contains the model and parameter values. |
| SimulationLog.txt | Example output file with simulation results. This file is created by the simulation upon execution. The name can be changed in the source. |
| CaveSmall.txt | Example cave layout file. These are read by the source code. This file refers to Layer 1 (L1) in text. |
| CaveLarge.txt | Example cave layout file. This file refers to Layer 2 (L2) in text. |
| fiReproxies User Manual | This manual. |

## Setup of the system

The main source file and the layout file can be stored in the different directories. In the source the variable areaFiles contains a list of all input cave layout files that are to be used, with full paths to where they are located. The directory where the results are stored is called workingDirectory. The user should first change these two variables to reflect the local file structure. The name of the output file is in the variable logFile.

## Layout of the cave area files

Each input file that describes a cave area consists of lines with integer values separated by commas. Each integer represents a grid cell. Each line must have an equal number of integers. Each integer can have three values: 0, 1, or 2. A value of 0 means that this grid cell is part of the cave wall. A value of 1 means inside the cave area, and a value of 2 means outside the cave. The border of the area must consist of at least three grid cells with 0 values.

As an example, the content of the CaveSmall.txt file (i.e., *Layer 1* in the main text) is given below:

0,0,0,0,0,0,0,0,0,0,0,0,0,0,0,0,0

0,0,0,0,0,0,0,0,0,0,0,0,0,0,0,0,0

0,0,0,0,0,0,0,0,0,0,0,0,0,0,0,0,0

0,0,0,0,0,0,0,1,0,0,0,0,0,0,0,0,0

0,0,0,0,0,0,1,1,1,0,0,0,0,0,0,0,0

0,0,0,0,0,0,1,1,1,0,0,0,0,0,0,0,0

0,0,0,0,0,1,1,1,1,1,0,0,0,0,0,0,0

0,0,0,0,0,1,1,1,1,1,1,0,0,0,0,0,0

0,0,0,0,0,1,1,1,1,1,1,0,0,0,0,0,0

0,0,0,0,0,1,1,1,1,1,1,0,0,0,0,0,0

0,0,0,0,0,1,1,1,1,1,1,0,0,0,0,0,0

0,0,0,0,0,1,1,1,1,1,1,0,0,0,0,0,0

0,0,0,0,1,1,1,1,1,1,1,1,0,0,0,0,0

0,0,0,0,1,1,1,1,1,1,1,1,1,0,0,0,0

0,0,0,0,1,1,1,1,1,1,1,1,1,0,0,0,0

0,0,0,0,0,2,2,2,2,2,2,2,0,0,0,0,0

0,0,0,0,0,0,2,2,2,2,2,0,0,0,0,0,0

0,0,0,0,0,0,0,0,0,0,0,0,0,0,0,0,0

0,0,0,0,0,0,0,0,0,0,0,0,0,0,0,0,0

0,0,0,0,0,0,0,0,0,0,0,0,0,0,0,0,0

# Running simulations

The simulation is written in R and the source file can be loaded into any R runtime environment, for instance into the RStudio environment. After changing the referred filenames (input layout files and working directory) the source can be executed.

When the source file is executed the following simulations are run:

- For all cave layout files, all simulation sessions are run
- The variable numberOfSessions defines how many sessions are run. In each session, all experiments are performed.
- The variable numberOfExperiments defines how many experiments are run. In each experiment, all number of occupations are simulated.
- The variable numberOfOccupations defines how many occupations are run. In each occupation, a number of fires are lit (variable numberOfFiresPerOccupation) and a number of lithic deposits (variable numberOfLithicsPerOccupation) deposited.
- After each experiment and each session the results are collected, printed and plotted, and subsequently stored in the allocated variables: experimentResults and sessionResultsMean and sessionResultsSD. After all simulations, the session results are written to the output file.

## Output of the simulations

Output consists of printed output on the screen, figures (graphs), density plots and an output file. Results are ordered per areaFile. Printed output consists of the percentage heated lithics per experiment per session, with each experiment having a given number of occupations. For every session the mean and standard deviation of all experiments are stored, and the values for all experiments in that session are plotted (experimentResults). Then the values of the mean of all experiment for every session is plotted (sessionResultsMean), including an indication of the standard deviation. See figure below.


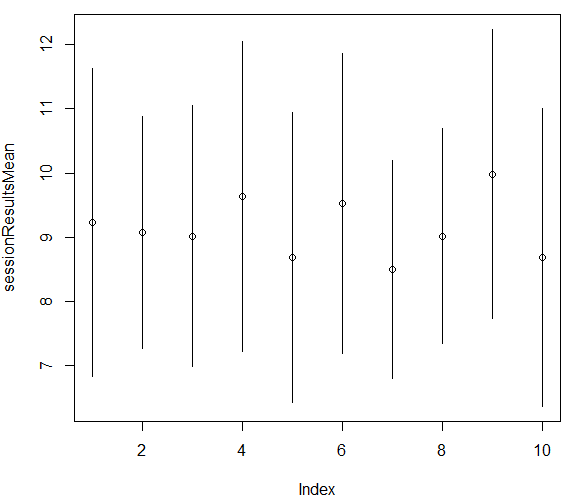


The output file contains the simulation results (mean and standard deviation per session) for all areafiles. An example is given below:


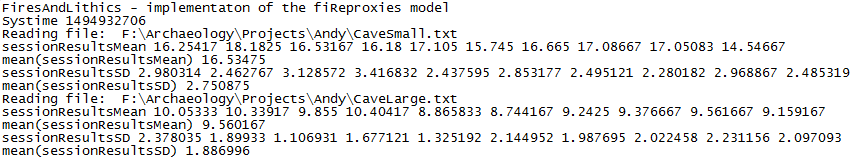


It is possible to display density plots. To do this the user must execute the levelplot commands in the code. These will produce density plots for fire occurrences, heated lithics and unheated lithics per occupation. Note: these are results per experiment, and the area maps are tilted sideways. An example for heated lithics is presented below, where more red means more heated lithics. Note that D1 is the most recent occupation (with therefore no heated lithics) and that thermal absorption is 0 (palimpsest situation).


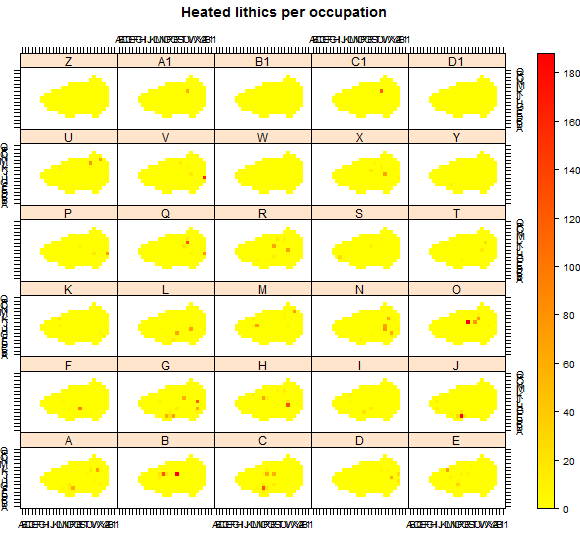


# Changing the model

The fiReproxies model aims to test certain hypotheses about fire and lithics deposition in cave areas. To facilitate this the user can change variables in the source code. These are referred to as model parameters and listed below. Lithics can variably be added to each occupation. The user may opt to distribute lithics either randomly or uniformly across the entire occupation surface, or as discrete scatters within selected grid cells. When deposited into a single grid cell there is some bleeding into neighboring grid cells. It is assumed that horizontal and vertical neighbors will receive 8% of the lithics, and diagonal neighbors 2% (these may be changed). The distribution is illustrated below. These percentages are hardcoded numbers in the source (from source code line 358 onwards). It is also assumed that a percentage (default 1%) of the deposited lithics is heated by being accidently introduced into a fire, regardless of the number of fires in the layer; this is coded in line 445.


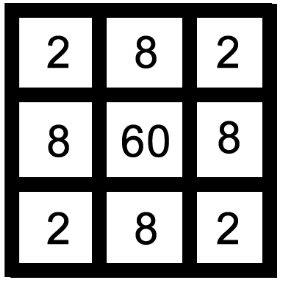


## Model parameters

The following parameters can be changed in the source code before running simulations.

| Parameter name | Default value | Description |
| --- | --- | --- |
| useFireLocationPreferences | false | If fires are located according to user defined preferences. If false, locations are chosen randomly. If true, the following three preferences are followed when placing fires. |
| useFireLocationNearPrevious | true | New fires are located on or next to fires from previous occupations. |
| useFireLocationNextToTheWall | false | If true fires are located next to a wall grid cell. If false, location is random. This variable is not used in this study. |
| useFireLocationOffTheWall | false | If true, new fires are located NOT next to the wall; if false, location is random. The variable is not used in this study. |
| useLithicLocationPreferences | true | If true, the following preference is followed when placing lithic deposits. If false, location is random but never directly on top of a fire in the same occupation. |
| useLithicLocationNextToTheFire | true | If true, lithics are located two grid cell away from a fire that is placed in this occupation (fires are placed first). There is thus always one grid cell between a scatter and the fire it was placed near to. |
| useLithicLocationRandom | false | If true, new lithics are randomly distributed throughout the area. The useLithicLocationPreferences setting is overruled (set to false). |
| useLithicLocationRandomUniform | true | If true, each cell receives an equal amount of lithic pieces: the number of pieces divided by the number of cells, rounded down. The remaining pieces are then distributed randomly. |
| numberOfLithicPieces  PerDistribution | 400 | The number of lithic pieces that is distributed (only used when useLithicLocationRandom is true). |
| percentageOfBurntLithicPieces  PerDistribution | 1 | Percentage of the distributed pieces of flint that is heated in each distribution (only used when useLithicLocationRandom is true). |
| useFireInNthLayersOnly | true | If false, all occupations have fires deposited. If true, only once in every n occupations (n defined by the next parameter) fires are placed. |
| fireInLayerN | 2 | If useFireInNthLayersOnly is true, only in every fireInLayerN occupation fires are deposited. If the value is 2, only in occupations 1,3,5,7, etc. fires are deposited. For a value of 10 the occupations with fires become 1,10,20, etc. |
| numberOfFiresPerOccupation | 1 | This is the number of fires deposited per occupation. |
| numberOfLithicsPerOccupation | 4 | The number of lithic scatters that are deposited per occupation. |
| fireSize | 1 | The number of grid cells a fire can occupy. Possible values are 1 and 4. With 4 a larger square of four grid cells will form one single fire event. |
| perc | 100 | The percentage of lithics that is heated in the occupations below the fire. This percentage is decreased for every one deeper occupation in the following code: perc = max(0, perc-10). This will decrease the amount of heated lithics with 10% for each occupation that is affected. The first has 90% heated, the second 80%, etc. In the source this variable is used and must be changed in multiple locations. |

## Simulation variables

Some variables within the source code define how many simulations are run, how many experiment must be performed and how many occupations per experiment occur. These can be adjusted by the user and are listed below:

| Variable name | Default value | Description |
| --- | --- | --- |
| areaFiles | Set of strings | A comma separated list of cave area filenames. For each file all simulations are executed. |
| numberOfSessions | 100 | Number of sessions. |
| numberOfExperiments | 30 | Number of experiments. |
| maxNumberOfOccupations | 30 | Maximum number of occupations, used for allocating memory for results. |
| numberOfOccupations | 30 | Number of occupations per experiment (cannot be more than maxNumberOfOccupations). |

# References

R Core Team, 2014. R: A language and environment for statistical computing. R Foundation for Statistical Computing, Vienna, Austria. URL <http://www.R-project.org/>.

RStudio Team, 2015. RStudio: integrated development for R. RStudio, Inc., Boston, MA. URL <http://www.rstudio.com>.
